# Exercise Ventilatory Inefficiency in Post-COVID-19 Syndrome: Insights from a Prospective Evaluation

**DOI:** 10.3390/jcm10122591

**Published:** 2021-06-11

**Authors:** Álvaro Aparisi, Cristina Ybarra-Falcón, Mario García-Gómez, Javier Tobar, Carolina Iglesias-Echeverría, Sofía Jaurrieta-Largo, Raquel Ladrón, Aitor Uribarri, Pablo Catalá, Williams Hinojosa, Marta Marcos-Mangas, Laura Fernández-Prieto, Rosa Sedano-Gutiérrez, Iván Cusacovich, David Andaluz-Ojeda, Blanca de Vega-Sánchez, Amada Recio-Platero, Esther Sanz-Patiño, Dolores Calvo, Carlos Baladrón, Manuel Carrasco-Moraleja, Carlos Disdier-Vicente, Ignacio J. Amat-Santos, J. Alberto San Román

**Affiliations:** 1 Departamento de Cardiología, Hospital Clínico Universitario de Valladolid, 47005 Valladolid, Spain; mcdeybarrafalcon@gmail.com (C.Y.-F.); mariog08@ucm.es (M.G.-G.); javitobar10@gmail.com (J.T.); caroliglesiasecheverria@gmail.com (C.I.-E.); ralaab@yahoo.es (R.L.); auribarrig@gmail.com (A.U.); pcatalaruiz@gmail.com (P.C.); williams_hinojosa@hotmail.com (W.H.); martamarcosma@outlook.com (M.M.-M.); amadarepla@yahoo.es (A.R.-P.); esanzpat@gmail.com (E.S.-P.); cbaladron@icicor.es (C.B.); manuelhcuv@gmail.com (M.C.-M.); ijamat@gmail.com (I.J.A.-S.); asanroman@secardiologia.es (J.A.S.R.); 2 Departamento de Neumología, Hospital Clínico Universitario de Valladolid, 47005 Valladolid, Spain; sofiajlargo@gmail.com (S.J.-L.); lfernandezpr@saludcastillayleon.es (L.F.-P.); rsedano@saludcastillayleon.es (R.S.-G.); blancadevegasanchez@gmail.com (B.d.V.-S.); cdisdier@separ.es (C.D.-V.); 3 Spain Centro de Investigación en Red de Enfermedades Cardiovasculares (CIBERCV), 28029 Madrid, Spain; 4 Departamento de Medicina Interna, Hospital Clínico Universitario de Valladolid, 47005 Valladolid, Spain; icusacovich@saludcastillayleon.com; 5 Departamento de Medicina Intensiva, Hospital Clínico Universitario de Valladolid, 47005 Valladolid, Spain; dandaluz@saludcastillayleon.es; 6 Grupo Emergente de Neumología Intervencionista SEPAR (GEBRYN), 28045 Madrid, Spain; 7 Instituto de las Ciencias de la Salud de Castilla y León (IECSCYL), 42002 Soria, Spain; 8 Departamento de Análisis Clínico, Hospital Clínico Universitario de Valladolid, 47005 Valladolid, Spain; mdcalvon@saludcastillayleon.es; 9 Centro de Investigación en Red de Enfermedades Respiratorias (CIBERES), 28029 Madrid, Spain

**Keywords:** post-COVID-19 syndrome, cardiopulmonary exercise testing, six-minute walking test, pulmonary function test, dyspnea, ventilatory inefficiency

## Abstract

Introduction: Coronavirus disease 2019 (COVID-19) is a systemic disease characterized by a disproportionate inflammatory response in the acute phase. This study sought to identify clinical sequelae and their potential mechanism. Methods: We conducted a prospective single-center study (NCT04689490) of previously hospitalized COVID-19 patients with and without dyspnea during mid-term follow-up. An outpatient group was also evaluated. They underwent serial testing with a cardiopulmonary exercise test (CPET), transthoracic echocardiogram, pulmonary lung test, six-minute walking test, serum biomarker analysis, and quality of life questionaries. Results: Patients with dyspnea (n = 41, 58.6%), compared with asymptomatic patients (n = 29, 41.4%), had a higher proportion of females (73.2 vs. 51.7%; *p* = 0.065) with comparable age and prevalence of cardiovascular risk factors. There were no significant differences in the transthoracic echocardiogram and pulmonary function test. Patients who complained of persistent dyspnea had a significant decline in predicted peak VO_2_ consumption (77.8 (64–92.5) vs. 99 (88–105); *p* < 0.00; *p* < 0.001), total distance in the six-minute walking test (535 (467–600) vs. 611 (550–650) meters; *p* = 0.001), and quality of life (KCCQ-23 60.1 ± 18.6 vs. 82.8 ± 11.3; *p* < 0.001). Additionally, abnormalities in CPET were suggestive of an impaired ventilatory efficiency (VE/VC_O2_ slope 32 (28.1–37.4) vs. 29.4 (26.9–31.4); *p* = 0.022) and high PET_CO2_ (34.5 (32–39) vs. 38 (36–40); *p* = 0.025). Interpretation: In this study, >50% of COVID-19 survivors present a symptomatic functional impairment irrespective of age or prior hospitalization. Our findings suggest a potential ventilation/perfusion mismatch or hyperventilation syndrome.

## 1. Introduction

Severe acute respiratory syndrome coronavirus 2 (SARS-CoV-2) is a highly virulent novel coronavirus and the cause of coronavirus disease 2019 (COVID-19). It triggers a strong immune response that becomes dysregulated and leads to systemic organ damage [1]. The estimated COVID-19 global mortality is 2.6% [2].

Most of the current knowledge of the disease has been directed toward the acute phase. Early reports during follow-up studies have reported that fatigue and dyspnea might affect up to 40% of COVID-19 survivors [3,4]. Furthermore, previous studies after hospital discharge have demonstrated abnormal pulmonary function tests in the early convalescent phase among COVID-19 survivors [5,6,7], with similar findings described after a three-month follow-up [8]. This fact suggests that there might be a great number of SARS-CoV-2 survivors presenting residual disabilities as has been demonstrated for alternative highly virulent coronaviruses [9,10].

To address this gap, the present study sought to explore the mid-term clinical course of COVID-19 survivors. We therefore described any clinical sequelae, persistent inflammatory parameters, pulmonary function, myocardial performance, and quality of life (QoL) with special emphasis on exercise capacity.

## 2. Materials and Methods

### 2.1. Study Design and Patient Selection

We conducted a single-center prospective study (NCT04689490) of patients with prior hospitalization because of COVID-19 who were admitted between March 2020 and April 2020. All eligible patients underwent a pre-specified follow-up 3 months after discharge with subsequent visits. A group of consecutive patients diagnosed with SARS-CoV-2 infection in the last fortnight of the study period who did not require hospital admission was also selected. Exclusion criteria were age < 18 years old, pregnancy, terminally ill patients, active SARS-CoV-2 infection, inability to exercise, and previous known severe cardiopulmonary disease.

All patients underwent a clinical assessment for symptom burden, evaluation of quality of life (QoL) with the Kansas City Cardiomyopathy Questionnaire (KCCQ) [11], venous blood sampling, resting echocardiography, six-minute walking test (6-MWT), tests of lung function (spirometry and diffusing capacity of the lungs for carbon monoxide), and treadmill cardiopulmonary exercise testing (CPET). All patients yielded a negative result in the reverse transcription polymerase chain reaction for SARS-CoV-2 48 h before the test of lung function and the CPET.

Patients were classified as post-discharge or ambulatory cohorts and subsequently as with or without dyspnea and compared. Patients were included in the dyspnea group if they reported New York Heart Association (NYHA) functional class ≥ II at the time of consultation.

The institutional local ethics committee approved the study protocol (CASVE PI-20-1894), and all patients provided written informed consent before inclusion. The work was carried out by following the guidelines of the Declaration of Helsinki of the World Medical Association.

### 2.2. Outcome Measure

The primary endpoint was self-reported functional capacity (reported as the NYHA) at 3 months after overcoming COVID-19, predicted peak oxygen consumption (V_O2_) according to CPET, and predicted carbon monoxide diffusion capacity (DL_CO_). Secondary endpoints included differences between (1) KCCQ score, (2) O2 pulse, (3) 6-MWT distance, (4) FEV1/FVC, (5) left ventricular ejection fraction (LVEF), and (6) inflammatory markers.

### 2.3. Clinical Laboratory Tests

We carried out all tests at a certified clinical laboratory (ISO 9001:2015). Ferritin and serum high-sensitivity C-reactive protein (hs-CRP) were measured by particle enhanced immunoturbidimetric and colorimetric assay, respectively (e501 Module Analyser^®^, Roche Diagnostics, Basel, Switzerland). Interleukin-6 (IL-6) was tested on IMMULITE^®^ 2000 immunoassay system (IMMULITE^®^ 2000 IL-6, Siemens Healthcare Diagnostic, Marburg, Germany). Quantification of biomarkers, such as the N-terminal prohormone of brain natriuretic peptide (NT-ProBNP) and the high-sensitivity T troponin (hs TnT) in plasma, were measured by electrochemiluminescence immunoassay with the analyzer Cobas^®^ 6000 c 601 (Roche Diagnostics, Basel, Switzerland). D-dimer was obtained by a turbidimetric test with the ACL Top 500^®^ hemostasis testing system (Werfen Company, Cuenca, Spain).

### 2.4. Resting Transthoracic Echocardiography

All patients underwent resting transthoracic echocardiography. All images were recorded in each of the standard projections in accordance with the recommendations of the American and European Societies of Echocardiography [12]. Images were analyzed offline using EchoPAC software (version 202) by two independent observers that determined valvular disease, myocardial deformation/strain (reported as global longitudinal strain), and diastolic function as well as right and left systolic ventricular function.

### 2.5. Pulmonary Function Test

Assessed pulmonary function tests were spirometry, lung volumes, and quantification of diffusion capacity for carbon monoxide (MasterScreen-Body/Diffusion; Sentry Suit v. 3.10) according to the recommendations of the European Respiratory Society [13,14]. At least three acceptable measurements were obtained. Recorded predicted parameters were forced expiratory volume in the 1st second (FEV1%), forced vital capacity (FVC%), FEV1/FVC ratio, residual volume (RV%), total lung capacity (TLC%), and diffusion capacity (DL_CO_% and K_CO_%).

### 2.6. Six-Minute Walking Test and Cardiopulmonary Exercise Test

The 6-MWT was performed according to standard methods [15]. In addition to the total distance and self-perceived exertion, pulse oxygen saturation and heart rate were recorded before and every minute until the test was completed.

All CPET were supervised by a physician and performed using a progressive incremental ramp protocol on a treadmill (Marquette MAX 1 treadmill, Marquette Electronics Inc., Milwaukee, WI, USA) integrated with a metabolic system (CPX Express, Medgraphics, Cardiorespiratory Diagnostic Systems, Medical Graphics Corporation, St. Paul, MN, USA) until patients complained of physical exhaustion or maximal capacity. During the procedure and recovery phase, there was continuous monitoring of the patient’s heart rhythm, peripheral oxygen saturation, blood pressure, and oxygen consumption (V_O2_). CPET was terminated in case of sudden arrhythmias, hypotension (or fall of systolic blood pressure >10 mmHg), repolarization abnormalities, or clinical symptoms suggestive of an underlying myocardial ischemia [16]. We did not exclude patients with a respiratory exchange ratio (RER) <1.05. As a measure of the aerobic capacity, we assessed predicted peak oxygen consumption (pV_O2_) and the anaerobic threshold (AT). To evaluate for ventilation/perfusion abnormalities, we also recorded the ventilatory equivalent of carbon dioxide (VE/VC_O2_) and partial pressure of end-tidal carbon dioxide (PET_CO2_) at AT.

### 2.7. Statistical Analysis

Categorical variables are reported as absolute values and percentages. Continuous variables are expressed as median (interquartile range (IQR)) or mean ± standard deviation (SD). The normality of continuous variables was verified with the Kolmogorov–Smirnov test and Q–Q plot. Categorical variables were compared with the chi-square test and the Fisher exact test when necessary. We compared continuous variables with the Student t-test or Mann–Whitney U test. A Spearman test was performed to analyze the correlation between CPET with the 6-MWT and lung function test. Scale scores of KCMQ were transformed to a 0–100 range by subtracting the lowest possible scale score, dividing by the range of the scale, and multiplying by 100. We performed the statistical analyses with the use of R software, version 3.6.1 (R Project for Statistical Computing) and IBM SPSS Statistics, Version 26.0. Armonk, NY: IBM Corp. Differences were statistically significant when the *p*-value was <0.05.

## 3. Results

In the study period, a total of 522 patients were admitted due to moderate–severe COVID-19 and 25% died [17]. A total of 53 patients met the inclusion criteria. In addition, 17 ambulatory patients were also included, leading to a final study population of 70 patients (see Supplementary Figure S1).

### 3.1. Main Baseline Characteristics and Predictors of Persistent Dyspnea

The main findings are listed in Table 1. The mean follow-up time of the second visit was 181 ± 42 days. Patients were subdivided into those with persistent dyspnea (n = 41, 58.6%) vs. asymptomatic (n = 29, 41.4%), with a greater rate of females (73.2 vs. 51.7%; *p* = 0.065) among those who complained of dyspnea. We did not observe any difference according to demographic variables and main comorbidities. Inpatients had a similar length of hospital stay and/or previous specific COVID-19 therapies. The need for hospital admission was not related to a greater rate of persistent dyspnea in the follow-up after multivariate adjustment analysis (data not shown).

### 3.2. Main Differences in KCCQ Score, Laboratory Parameters, and Echocardiographic Findings According to the Presence of Persistent Dyspnea

**Figure 1:**
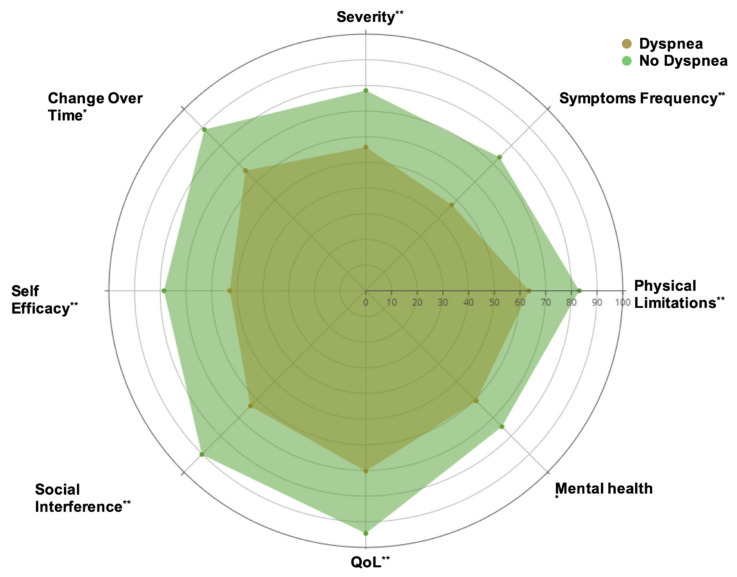
Quality of life assessment with Kansas City Cardiomyopathy Questionnaire (KCMQ). * (*p* < 0.01) and ** (*p* < 0.001) indicate significant differences.

**Figure 2:**
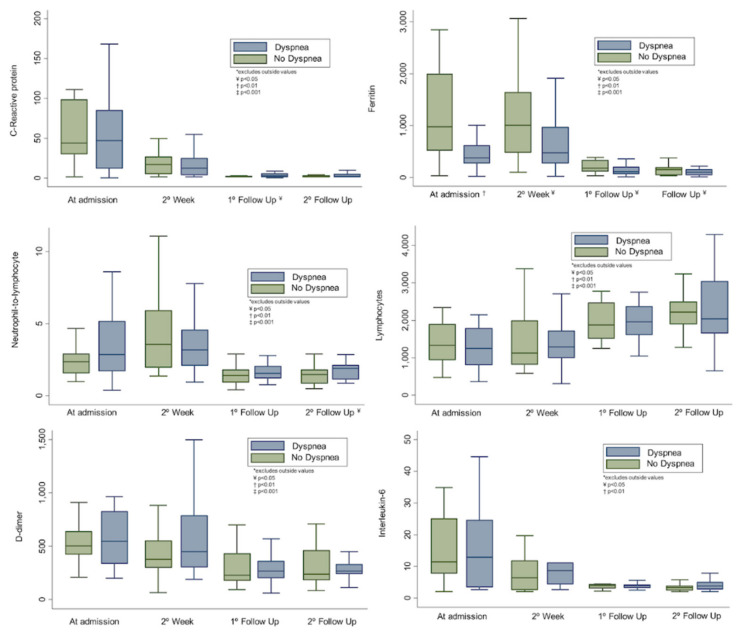
Temporal dynamic changes of inflammatory markers and lymphocytes from hospital admission to follow-up in the hospitalized cohort. * Excludes outside values; ¥ (*p* < 0.05); † (*p* < 0.01); ‡ (*p* < 0.001).

Patients with persistent dyspnea presented lower global KCCQ scores, both in the physical and emotional domains (*p* < 0.001) (see Figure 1). There were no significant differences (*p* > 0.05) in hemoglobin (14 (13.1–15.2) vs. 14.2 (13.7–16) g/dL), hs-CRP (1.75 (1–4.25) vs. 1.2 (1–2.15) mg/L), IL-6 (3.6 (2.6–4.7) vs. 3.2 (2.5–3.7) pg/mL), ferritin (94.3 (46.1–142.1) vs. 145.3 (51.6–181.2) ng/mL), D-dimer (268 (221–352) vs. 246 (180–384) ng/mL), and NT-ProBNP (37 (19.5–55.4) vs. 65 (29–127) pg/mL) in either group, with the exception of the neutrophil-to-lymphocyte ratio (1.8 (1.17–2.12) vs. 1.32 (0.98–1.76); *p* = 0.022). A detailed summary of all parameters and some additional parameters are summarized in Table 2. Notably, inpatients showed a trend towards normalization (from hospital admission to follow-up) of all the inflammatory indices (hs-CRP, IL-6, ferritin, and D-dimer) and lymphocyte count (see Figure 2), irrespective of the persistence of symptoms.

Resting echocardiography findings among COVID-19 survivors are summarized in Table 2. The left ventricular systolic and diastolic functions were comparable between the study groups. No severe valvular heart disease was observed, only mild mitral regurgitation was presented in 11 patients (19.5 vs. 10.3%; *p* = 0.342) and mild aortic regurgitation in 6 patients (12.2 vs. 3.4%; *p* = 0.389). In addition, all patients had a normal right ventricular function and no indirect signs of pulmonary hypertension, though symptomatic patients had a greater estimated right ventricular pressure (22 (18–26) vs. 18 (12–19) mmHg; *p* = 0.020).

### 3.3. Cardiac and Pulmonary Function Test

A summary of the results from the cardiopulmonary evaluation is reported in Table 2. Overall, there were no differences in respiratory mechanics at follow-up. Whereas patients with persistent dyspnea showed a trend towards a lower predicted DL_CO_ (84 (70–92.1) vs. 91 (85–102); *p* = 0.098).

During CPET, compared with asymptomatic controls, patients with persistent dyspnea presented lower predicted pV_O2_ (77.8 (64–92.5) vs. 99 (88–105); *p* < 0.001) and pV_O2_ at AT (13.6 (9.2–17) vs. 18.3 (15.2–19.5); *p* = 0.003). Both groups presented similar RER (1.08 (1.05–1.16) vs. 1.13 (1.05–1.28); *p* = 0.172) and % of predicted O_2_ pulse (98 (73–110) vs. 106 (96–110); *p* = 0.054). Regarding the ventilatory efficiency, a higher VE/V_CO2_ slope (32 (28–37.4) vs. 29.4 (26.9–31.4); *p* = 0.022) and lower PET_CO2_ at AT (34.5 (32–39) vs. 38 (36–40); *p* = 0.025) in patients with persistent dyspnea were detected. Neither desaturation on exercise nor differences in breathing reserve were detected. Blood pressure was comparable at any given moment but peak heart rate (87 (79.3–94.5) vs. 95 (88–100); *p* = 0.003) was lower in symptomatic patients although within normal values.

Moreover, symptomatic patients achieved shorter distances in the 6-MWT (535 (467–600) vs. 611 (550–650) m; *p* = 0.001), presenting a positive correlation with pV_O2_ (R = 0.533; *p* < 0.001) in the global study population. No patient presented oxygen desaturation during the 6-MWT. The main findings are summarized in Table 2.

### 3.4. Hospitalized and Ambulatory Patients

**Figure 3:**
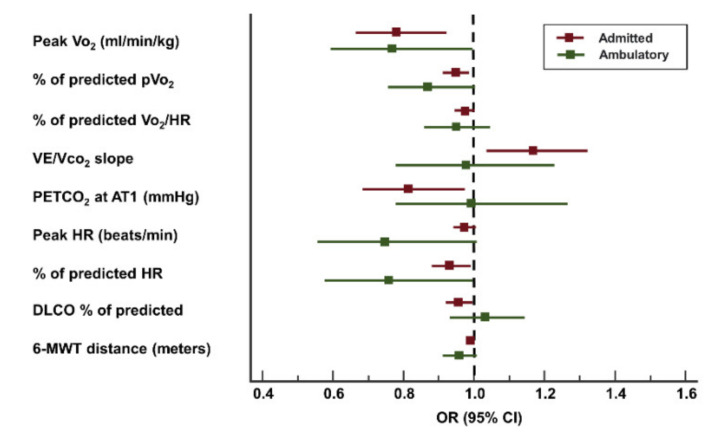
Predictors of dyspnea among hospitalized and ambulatory patients.

Outpatient clinical and main hospitalized clinical and functional characteristics are shown in Supplementary Tables S1 and S2. We did not observe differences in the main baseline characteristics. On the contrary, irrespective of the previous history of hospitalization, symptomatic patients presented a significantly lower exercise tolerance compared to their homologs in terms of predicted pVo2 and total distance in the 6-MWT. We also observed a lower QoL in the KCCQ in both groups. The main features associated with persistent dyspnea, both in ambulatory and hospitalized patients, are presented in Figure 3.

### 3.5. Summary of Published Evidence

We also summarized all the current data regarding persistent symptoms after acute COVID-19 (see Table 3). According to the available evidence [3,4,5,6,7,8,18,19,20,21,22,23,24,25,26], the most frequent symptoms during follow-up are dyspnea and fatigue. Six studies evaluated pulmonary function, detecting a decrease in DL_CO_ with a normal FEV1/FVC in the global post-COVID-19 population at short-term follow-up [5,6,7,8,24]. Two studies evaluated the functional status with the 6-MWT [5,21,24], suggesting a decrease in functional capacity. One study reported data about outpatients without prior hospitalization in early convalescence, the most common persisting symptoms were fatigue, dyspnea, and cough [27]; no functional tests were performed in this setting.

## 4. Discussion

This single-center prospective study evaluated persistent dyspnea throughout analyses of patients with prior history of SARS-CoV-2 infection. The main findings are as follows: (1) more than half of the patients complained of persistent dyspnea in the mid-term follow-up irrespective of the need for hospital admission and despite healed infection and normalization of inflammatory markers; (2) these subjective symptoms presented objective translation as reduced QoL (KCCQ) and exercise performance (6-MWT and CPET); and (3) conversely, the indices of cardiac and ventilatory inefficiency measured during CPET suggested a potential ventilation/perfusion mismatch.

### 4.1. Rationale for Post-COVID-19 Symptom Persistence

Persistent symptoms were more common in women but not in elderly patients. Although in our study 7% of the patients required ICU admission, the persistence of symptoms was related neither to ICU nor to hospital admission. The high rate of post-COVID-19 symptomatic patients is in agreement with alternative coronavirus outbreaks. Although several studies have explored the symptom burden [3,4,5,6,7,8,18,19,20,21,22,23,24,25,26], similarly reporting a substantial proportion of patients with persistent dyspnea and fatigue, limited information exists regarding exercise capacity [5,6,7,8,24]. Two prior studies have reported a decreased 6-MWT distance among survivors [5,21,24]. Such findings, as well as the lower scores on the QoL questionnaire detected in our research, can vary depending on several conditions [11] and therefore have a limited prognostic value in this context. The significantly lower exercise capacity (measured as predicted pV_O2_) in the CPET has been previously related to increased mortality in alternative contexts [16,28].

The CPET data in symptomatic patients are compatible with ventilatory inefficiency (high VE/V_CO2_ with a lower PET_CO2_) strongly suggestive of a ventilation/perfusion mismatch due to pulmonary vasculopathy [29] and also supported by a mild reduction of DL_CO_ already described by other authors [5,6,7,8,24]. This pulmonary vascular disease, according to our findings, does not seem to have a component of cardiac contribution, persistent inflammation, iron deficiency, or air-flow limitation. Despite the very limited shreds of evidence from autopsy reports, several factors are supporting this vascular mechanism. First, endothelial injury has been widely described in SARS-CoV-2 infected patients, explaining multiorgan affection [30]. Second, postmortem analyses have reported diffuse alveolar damage and small microthrombi in pulmonary capillaries as the most characteristic findings [31,32]. Third, intravascular fibrin aggregates in pulmonary vessels were the most common finding regardless of the type of pulmonary injury [33]. Fourth, endothelial dysfunction has been linked before to activation of the coagulation cascade in COVID-19 patients [30]. Fifth, intussusceptive angiogenesis was also observed in lungs from COVID-19 patients [32], which may, in theory, disrupt the structure of the microcirculation and has been previously described in chronic thromboembolic pulmonary hypertension [34].

Could the persistence of microvascular thrombus be the hallmark of post-COVID-19 dyspnea? This hypothesis could be supported by the fact that 42.5% of our study population underwent computed tomography and 41% compression venous ultrasonography during follow-up without any evidence of thrombi in the pulmonary or femoral vessels. Besides, Ong et al. reported a decreased exercise capacity amongst SARS-CoV survivors that was not explained by pulmonary or ventilatory function [35]. Similarly, Jelle et al. proposed that restriction among COVID-19 survivors did not explain the residual symptoms, and low DL_CO_ was not explained either by anemia [24]. It thus appears that circulatory impairment might be the missing link that could explain some of the residual symptoms in post-COVID-19 syndrome, but we should keep in mind that the mechanism remains obscure. The ventilatory inefficiency observed in our symptomatic cohort is also observed in the hyperventilation syndrome [36], which has been recently suggested as a potential mechanism of the post-COVID-19 syndrome [37]. In fact, this syndrome has been described in broad lung conditions, such as acute pulmonary embolism, pneumonia, chronic interstitial lung diseases, and after viral infections [36].

Motiejunaite et al. reviewed eight patients with residual symptoms after COVID-19 infection and reported similar findings to us. All of them showed a decreased exercise capacity, and five patients showed an elevated VE/V_CO2_ ratio suggestive of hyperventilation syndrome [37]. In this respect, hyperventilation may induce hypocapnia and it is believed to be caused by an abnormal ventilatory control by either stimulation of activator systems or suppression of inhibitory systems. This hyperventilation-induced hypocapnia could explain the persisting exercise intolerance and CPET pattern observed in these patients. However, Crisafulli et al. evaluated patients with persistent hypocapnia and did not observe an increased rate of persistent dyspnea or fatigue, but it was associated with impaired diffusion [38]. Because both can present with a similar pattern in CPET [36], future studies with ventilation/perfusion scanning are warranted.

Finally, another potential mechanism could be the autonomic dysfunction. Maladaptive function of the autonomic nervous system in COVID-19 survivors may contribute to the persistence of fatigue, shortness of breath, and palpitations [37]. Consistent with this hypothesis, Dorelli et al. observed a slower heart rate recovery suggestive of dysautonomia among post-COVID-19 patients with ventilatory inefficiency [39]. The cause of this autonomic dysfunction is not clear but probably involves infection-mediated endothelial injury that causes an abnormal activation of the parasympathetic nervous system.

### 4.2. Prognostic Implications

The aforementioned impaired functional parameters suggest that post-COVID-19 patients with persistent symptoms should undergo dedicated follow-up programs. Not surprisingly, these findings are also similar to those reported in the previous coronaviruses during early convalescence and long-term follow-up [9,10,35]. Persistent symptoms amongst outpatients are not rare [22,27]. According to our findings, a low aerobic capacity and QoL could be a constant among patients developing this kind of sequalae, irrespective of the clinical course during the infection. Nonetheless, the mechanism and the prognostic relevance of these findings remain unclear. Whether life expectancy might be modified due to post-COVID-19 syndrome is a question yet to be answered, as similar findings in other cardiac or respiratory conditions are considered predictors of mortality [16,28,39].

### 4.3. Limitations

Despite the prospective nature of the present study, the main limitation is the relatively modest sample size from a single-center and the lack of pre-COVID-19 cardiopulmonary functional tests; thereby, we cannot rule out that previous non-diagnosed chronic conditions may interfere despite the strict inclusion criteria. Second, patients did not undergo CPET with invasive hemodynamic monitoring, arterial blood gas analysis, or exertional echocardiography, even though it might have been more accurate to evaluate exercise-induced dyspnea. Third, we chose a heterogeneous sample to mimic a more real-world clinical scenario, however, it is also a potential limitation that limits its generalizability. Fourth, a potential selection bias cannot be ruled out, as symptomatic patients may be more predisposed to collaborate or seek early medical attention. Finally, we cannot draw causal inferences based on our study results, which should be considered hypothesis-generating. Future prospective studies should be encouraged to validate and properly understand if the reported data are the result of COVID-19.

## 5. Conclusions

In this study, >50% of COVID-19 survivors present a symptomatic functional impairment irrespective of age or prior hospitalization. Our findings are consistent with a perfusion/ventilation mismatch that likely reflects gas exchange inefficiency or hyperventilation syndrome. On this basis, systematic follow-up of patients with persistent symptoms following SARS-CoV-2 infection, including cardiopulmonary functional tests, should be encouraged at longer term follow-up.

## Figures and Tables

**Table 1:** Baseline characteristics and main features during mid-term follow-up in recovered COVID-19 patients according to the presence of persistent dyspnea.

Variable	All Population n = 70	Persistent Dyspnea * n = 41 (58.6)	No Residual Dyspnea n = 29 (41.4)	*p*-Value
**Demographics, anthropometric data, and comorbidities**				
Female sex	45 (64.3)	30 (73.2)	15 (51.7)	0.065
Age (years)	54.8 ± 11.9	54.9 ± 10.5	54.6 ± 13.9	0.914
BMI (kg/m^2^)	27.2 ± 4.6	28 ± 4.9	26 ± 3.9	0.067
BSA (m^2^)	1.82 ± 0.18	1.81 ± 0.18	1.84 ± 0.18	0.423
CKD **	3 (4.4)	3 (7.3)	0	0.271
Diabetes	3 (5.9)	3 (10.3)	0	0.249
Dyslipidemia	13 (19.1)	7 (17.1)	6 (22.2)	0.597
Hypertension	18(26.5)	12 (29.3)	6 (22.2)	0.519
Hypothyroidism	11 (16.2)	7 (17.1)	4 (14.8)	0.999
IHD	1 (1.5)	0	1 (3.7)	0.397
Prior pulmonary disease	5 (7.4)	3 (7.3)	2 (7.4)	0.999
Prior rheumatic disease	4 (7.5)	4 (9.8)	0	0.146
Prior stroke/TIA	1 (1.5)	1 (2.4)	0	0.999
**Treatment during hospitalization †**				
LOS (days)	8(6–11.5)	8 (6–11)	8 (6–13)	0.954
Anticoagulation	7 (13.2)	3 (9.7)	4 (18.2)	0.703
Azithromycin	49 (92.5)	29 (93.5)	20 (90.9)	0.999
Hydroxychloroquine	50 (94.3)	28 (90.3)	22 (100)	0.258
Glucocorticoids	29 (54.7)	15 (48.4)	14 (63.6)	0.272
Lopinavir/Ritonavir	51 (96.2)	29 (93.5)	22 (100)	0.505
Statins	4 (7.8)	2 (6.9)	2 (9.1)	0.999
**Symptoms during follow-up**				
KCCQ summary score	70.0 ± 19.4	60.1 ± 18.6	82.8 ± 11.3	<0.001
Chest pain	8 (11.4)	7 (17.1)	1 (3.4)	0.128
Fatigue	20 (28.6)	17 (41.5)	3 (10.3)	0.005
Headache	10 (14.3)	6 (14.6)	4 (13.8)	0.999
Myalgia	6 (9.8)	4 (0.8)	2 (6.9)	0.999
Neurological symptoms ‡	14 (20)	5 (12.2)	9 (31)	0.052
Palpitations	10 (14.3)	6 (14.6)	4 (13.8)	0.999

Abbreviations: CKD: chronic kidney disease; IHD: ischemic heart disease; KCCQ: Kansas City Cardiomyopathy Questionnaire; TIA: transient ischemic attack; LOS: length of stay. * Persistent dyspnea was defined as NYHA ≥ II. ** Chronic kidney disease was defined as a glomerular filtration rate of <60 mL/min or need for dialysis. † Only applies to those with prior hospitalization. ‡ Includes paresthesia, olfactory, and taste abnormalities. Values are median (IQR), mean ± SD, or n (%). Bold indicates significative differences (*p* < 0.05).

**Table 2:** Echocardiography, cardiopulmonary exercise test during, pulmonary function test during mid-term follow-up in recovered COVID-19 patients.

	All Population n = 70	Persistent Dyspnea * n = 41 (60)	No residual Dyspnea n = 29 (40)	*p*-Value
**Laboratory markers**				
Albumin (g/L)	4.5 (4.4–4.7)	4.5 (4.4–4.7)	4.5 (4.4–4.6)	0.177
AST (UI/L)	19 (16–25)	21 (17–25)	17 (13–22)	0.054
C-reactive protein (mg/L)	1.3 (1–2.8)	1.75 (1–4.25)	1.2 (1–2.15)	0.173
Creatinine (mg/dL)	0.84 (0.75–0.98)	0.82 (0.76–0.98)	0.85 (0.75–0.97)	0.995
D-dimer (ng/mL)	265 (188–377)	268 (221–352)	246 (180–384)	0.581
Ferritin (ng/mL)	113.1 (50.1–159.1)	94.3 (46.1–142.1)	145.3 (51.6–181.2)	0.063
Interleukin-6 (pg/mL)	3.42 (2.6–4.4)	3.6 (2.6–4.7)	3.2 (2.5–3.7)	0.174
Hemoglobin (g/dL)	14 (13.5–15.3)	14 (13.1-15.2)	14.2 (13.7–16)	0.107
Lymphocytes (cells/mm^3^)	2,185 (1800–2790)	2200 (1660–2790)	2170 (1850–2510)	0.638
Neutrophil/Lymphocyte	1.54 (1.08–2.04)	1.8 (1.17–2.12)	1.32 (0.98–1.76)	0.022
NT-ProBNP (pg/mL)	41 (23–68)	37 (19.5-55.4)	65 (29–127)	0.051
Hs TnT (pg/mL)	5.4 (3.1–7.54)	5.5 (3.2–7)	5.3 (3.2–9.6)	0.504
TSH (mU/L)	2.05 (1.68–3.24)	2.11 (1.66–3.4)	1.97 (1.7–2.69)	0.722
**Resting echocardiographic findings**				
LAVI (mL/m^2^)	22.1 (17.7–27.8)	21.2 (18.3–30)	22.5 (17.7–26.1)	0.740
LVEF (%)	64 (59–68)	65 (59–68)	63 (60–69)	0.962
LVEDVi (ml/m^2^)	75 (66–100)	41.2 (36.2–50.6)	45.3 (40.5–54.2)	0.123
LVESVi (ml/m^2^)	16.2 (12.3–20.1)	14.1 (12.4–21)	16.7 (14–21)	0.194
Mitral E/A ratio	0.9 (0.76–1.22)	0.89 (0.79–1.19)	0.93 (0.75–1.27)	0.697
Mitral e’ lateral	0.11 (0.09–0.14)	0.8 (0.09–0.13)	0.11 (0.09–0.11)	0.822
Average E/e´ ratio	6.5 (4.9–7.9)	6.6 (4.9–8.9)	6.2 (5–7.3)	0.284
TAPSE (mm)	23 (20–26)	23 (20–27)	23 (22–25)	0.472
S’ (cm/sec)	13 (12–15)	13 (12–14.5)	13 (12–15)	0.392
RVSP (mmHg)	19 (15–24)	22 (18–26)	18 (12–19)	0.020
Global longitudinal strain (%)	20 (22–19)	20 (22–19)	20 (22–19)	0.806
**Cardiopulmonary exercise test**				
Breathing reserve (%)	41 (32–51)	46 (30–54)	40 (36–46)	0.319
RER	1.11 (1.05–1.21)	1.08 (1.05–1.16)	1.13 (1.05–1.28)	0.172
Peak Vo_2_ (ml/min/kg)	19.4 (17.2–24.8)	17.8 (15.8–21.2)	22.8 (18.8–27.7)	<0.001
% of predicted pVo_2_	88 (76–100)	77.8 (64-92.5)	99 (88–105)	<0.001
Vo_2_ at AT_1_ (ml/min/kg)	15.4 (12–19.2)	13.6 (9.2–17)	18.3 (15.2–19.5)	0.003
% of predicted Vo_2_ /HR	101 (83–110)	98 (73–110)	106 (96–110)	0.054
VE/Vco_2_ slope	30.3 (27.5–34.9)	32 (28.1–37.4)	29.4 (26.9–31.4)	0.022
VE/Vco_2_ at AT_1_	34.7 (32.3–39.5)	37.2 (31.5–42.3)	33.7 (32.5–36.4)	0.194
PETCO_2_ (mmHg) at AT_1_	38 (33.5–39.5)	34.5 (32–39)	38 (36–40)	0.025
Resting HR (beats/min)	79 (71–85)	78 (70–80)	80 (74–86)	0.357
Peak HR (beats/min)	155 (140–163)	148 (140–159)	161 (147–169)	0.018
% of predicted HR	90.3 (83.9–97.4)	87 (79.3–94.5)	95 (88–100)	0.003
Resting O_2_ saturation (%)	97 (96–98)	97 (96–98)	97 (96–98)	0.620
Peak O_2_ saturation (%)	97 (96–98)	97 (96–98)	97 (96–98)	0.388
Resting systolic BP (mmHg)	139 (124–146)	140 (125–150)	123 (134–142)	0.205
Peak systolic BP (mmHg)	143 (160–177)	155 (139–175)	160 (151–177)	0.319
Resting diastolic BP (mmHg)	86 (77–95)	90 (80–97)	82 (75–89)	0.034
Peak diastolic BP (mmHg)	90 (81–100)	90 (82–106)	91 (80–95)	0.443
**Pulmonary lung function**				
DL_CO_ % of predicted	88.8 (80–97)	86 (74.5-95.3)	90 (83.5–100)	0.098
K_CO_ % of predicted	95.3 (88.7–109)	94.6 (86.5–107)	96 (89–110.5)	0.493
FEV1 % of predicted	112 (103.5–121.5)	113 (102–122)	115 (105–124)	0.690
FVC % of predicted	116 (105–131)	115 (104–132.5)	116 (108.5–120)	0.989
FEV1/FVC (%)	100 (91.6–105)	98.5 (86.5–106)	102 (97–104)	0.466
RV % of predicted	101 (89.8–118.5)	106.5 (94.3–119)	95 (85–109)	0.138
TLC % of predicted	100 (96.5–111)	100 (96–112.7)	101 (97–109)	0.801
6-MWT distance (meters)	558 (500–615)	535 (467–600)	611 (550–650)	0.001

Abbreviations: 6-MWT: six-minute walking test; AT: anaerobic threshold; DLCO: carbon monoxide diffusion capacity; E/e’: ratio of early diastolic mitral inflow velocity to early diastolic mitral annulus velocity; FEV1: forced expiratory volume in 1 min; FVC: forced vital capacity; HR: heart rate; LAVI: left atrial volume indexed; LVEF: left ventricular ejection fraction; LVEDVi: left ventricular end-diastolic volume indexed; LVESVi: left ventricular end-systolic volume indexed; METs: metabolic equivalents; RV: residual volume; TLC: total lung capacity. * Persistent dyspnea was defined as NYHA ≥ II. Values are median (IQR). Bold indicates significative differences (*p* < 0.05).

**Table 3:** Summary of the available data of persistent symptoms after acute COVID-19.

First Author	Journal/Year	Design	Number of Patients	Timing of Assessment	Clinical Findings	Biomarkers	Functional Findings	QoL Assessment
Garrigues et al. [3]	*J. Infection*/2020	Single-center Prospective	120	>3 months	Dyspnea 41.7% Fatigue 55%	Not reported	Not reported	Yes
Carfi et al. [4]	*Jama*/2020	Single-center Prospective	143	2 months	Dyspnea 43.4% Fatigue 53.1%	Not reported	Not reported	Yes
Huang et al. [5]	*Eur. Respir. J.*/2020	Single-center Retrospective	57	1 month	Dyspnea 7% Cough 10.5%	CRP 9.7 ± 13.8 LDH 175.5 ± 43.6 Lymphocytes 1.6 ± 0.5	6-MWD 562 ± 45.3 FEV1/FVC 81.2 ± 6.1 DL_CO_ 78.4 ± 3.6	No
Frija-Masson et al. [6]	*Eur. Respir. J.*/2020	Single-center Retrospective	50	1 month	(Only assessed asymptomatic)	Not reported	FEV1/FVC 81 (75–87) DL_CO_ 80 (70–92) K_CO_ 94 (78–108)	No
Mo et al. [7]	*Eur. Respir. J.*/2020	Single-center Retrospective	110	Hospital discharge	(Evaluated on the day or day after discharge)	Not reported	FEV1/FVC 80.7 ± 5.81 DL_CO_ 78.2 ± 14.3 K_CO_ 92.1 ± 16.7	No
Zhao et al. [8]	*Eclinicalmedicine*/2020	Multi-center Retrospective	55	>3 months	Dyspnea 14.5% Fatigue 16.4%	D-dimer 230 vs. 420 Lymphocyte 1.42 vs. 1.22	Abnormal pulmonary function 14 patients	No
Carvalho-Schneider et al. [18]	*Clin. Microbiol. Infect.*/2020	Single-center Prospective	150	1 and 2 months	Dyspnea 10.7% and 7.7% Chest pain 18% and 13% Flu-like 36% and 21%	Not reported	Not reported	No
Rosales-Castillo et al. [19]	*Med. Clin. (Barc.)*/2020	Single-center Retrospective	118	>1 month	Dyspnea 31.4% Fatigue 30.5%	Not reported	Not reported	No
Mandal et al. [20]	*Thorax*/2020	Single-center Prospective	384	> 1month	Dyspnea 53% Fatigue 69% Cough 34%	CRP 1 (1–4) D-dimer 384 (242–665) Lymphocytes 1.94 (1.44–2.52)	Not reported	No
Daher et al. [21]	*Respir. Med.*/2020	Single-center Prospective	33	6 weeks	Dyspnea 33% Fatigue 45% Cough 33%	CRP 2 (1.1–7.9) LDH 213 (196–227) Ferritin 154.6 (82–364) NT-ProBNP 183 (43–474) Hs Troponin-T 8 (4–21)	6-MWD 380 (180–470) FEV1/FVC 79 (76–85) DL_CO_ 65 (53–73) K_CO_ 77 (69–95) LVEF 52 (50–52)	Yes
Göertz et al. [22]	*ERJ Open Res.*/2020	Multi-center Prospective	2113	3 months	Dyspnea 71% Fatigue 87% Cough 38%	Not reported	Not reported	No
Xiong et al. [23]	*Clin. Microbil. Infect.*/2020	Longitudinal study	538	3 months	Dyspnea 21% Fatigue 28.3%	Not reported	Not reported	No
Jelle et al. [24]	*Respir. Med.*/2020	Cross-sectional	220	10 weeks	Dyspnea 47% Fatigue 66%	Not reported	38% with restrictive pulmonary function and low DL_CO_ in 22%	No
Tabada et al. [25]	*J. Infection*/2020	Cross-sectional	183	6 months	Dyspnea 10.9%	Not reported	Not reported	Yes
McCue et al. [26]	*Intensive Care Med.*/2020	Not reported	30	12–16 weeks	Pain 67%	Not reported	Not reported	Yes
Tenforde et al. [27]	*MMWR Morb. Mortal. Wkly. Rep.*/2020	Not reported	175	2–3 weeks	Dyspnea 26% Fatigue 35% Cough 43%	Not reported	Not reported	No

Abbreviations: 6-MWT: six-minute walking test; CRP: C-reactive protein; DLCO: carbon monoxide diffusion capacity; FEV1/FVC: forced expiratory volume in 1 min/forced vital capacity ratio; LDH: lactate dehydrogenase; LVEF: left ventricular ejection fraction.

## Data Availability

The data presented in this study are available upon reasonable request from the corresponding author. The data are not publicly available due to them containing information that could compromise the privacy of the research participants.

## Supplementary Materials

The following are available online at https://www.mdpi.com/article/10.3390/jcm10122591/s1, Table S1: Baseline characteristics and main features during mid-term follow-up in recovered COVID-19 patients according to the presence of persistent dyspnea in outpatients, Table S2: Baseline characteristics and main features during mid-term follow-up in recovered COVID-19 patients according to the presence of persistent dyspnea among hospitalized patients, Figure S1: Schematic flowchart of the recovered COVID-19 patients included in the study, showing main findings.

## Abbreviations

6-MWT	Six-minute walking test
COVID-19	Coronavirus disease 2019
CPET	Cardiopulmonary exercise test
DL_CO_	Carbon monoxide diffusion capacity
KCCQ	Kansas City Cardiomyopathy Questionnaire
PET_CO2_	Partial pressure of end-tidal carbon dioxide
QoL	Quality of Life
SARS-CoV-2	Severe acute respiratory syndrome coronavirus 2
VE/V_CO2_	Ventilatory equivalent of carbon dioxide
V_O2_	Oxygen consumption
