# Supplementary material for: Exercise Ventilatory Inefficiency in Post-COVID-19 Syndrome: Insights from a Prospective Evaluation
Source: J Clin Med. 2021 Jun 11;10(12):2591. doi: 10.3390/jcm10122591 (PMC8230788; doi:10.3390/jcm10122591)
Supplement: Supplementary file 1 [file jcm-10-02591-s001.zip › jcm-1229150-supplementary.pdf]

Supplementary Materials

Suppl. Table 1. Baseline characteristics and main features during mid-term follow-up in recovered COVID-19 patients according to the presence of persistent dyspnea in outpatients.

Suppl. Table 2. Baseline characteristics and main features during mid-term follow-up in recovered COVID-19 patients according to the presence of persistent dyspnea among hospitalized patients.

Suppl. Figure 1. Schematic flowchart of the recovered COVID-19 patients included in the study, showing main findings.

Suppl. Table S1. Baseline characteristics and main features during mid-term Figure 19. patients according to the presence of persistent dyspnea in outpatients.

	**Persistent Dyspnea*<br>n = 10 (59)**	**No residual dyspnea<br>n= 7 (40)**	**p-value**
**Demographics, comorbidities, and symptoms**			
Female sex (%)	6 (85)	8 (80)	0.999
Age (years)	47.5 (38–57)	46 (36–51)	0.406
BSA (m^2)	1.86 (1.69–1.94)	1.69 (1.67–1.74)	0.187
Dyslipidemia (%)	1 (10)	1 (14)	0.999
Hypertension (%)	1 (10)	0	0.999
KCCQ summary score	48.8±7.1	89.4±6.5	**<0.001**
Fatigue (%)	5 (50)	0	**0.044**
Myalgia (%)	3 (30)	0	0.228
Neurological symptoms (%)	2 (28.6)	3 (30)	0.999
**Resting echocardiographic findings**			
LAVI (mL/m^2)	21.2 (20.5–26)	21.3 (17.7–23)	0.848
LVEF (%)	59 (58–70)	64 (62–68.5)	0.630
Mitral E/A ratio	1.2 (0.89–1.8)	1.4 (1.23–1.71)	0.368
Average E/e′ ratio	6.2 (4.64–6.6)	6 (5.6–7.2)	0.832
TAPSE (mm)	20.5 (20–26)	22 (21–27)	0.237
Global longitudinal strain (%)	22 (22.5–21)	22.5 (23–21)	0.471
**Cardiopulmonary exercise test**			
Peak Vo_2 (ml/min/kg)	20.3 (17.2–23.2)	29.5 (24.5–33.2)	**0.023**
% of predicted pVo_2	82 (76–94)	103 (101–108)	**0.011**
Vo_2 at AT_1 (ml/min/kg)	15.8 (15.2–19.8)	22.2 (18.7–25.3)	0.142
Vo_2/HR	10 (8.4–9.2)	10.2 (9.8–11.8)	0.427
VE/Vco_2 slope	27.6 (23.6–29.8)	29.7 (27.1–30.7)	0.672
$PETCO_2$ at AT_1 (mmHg)	40 (35–43)	38 (38–41)	0.948
% of predicted HR	85.6 (80.8–88.8)	95 (92.6–96)	**0.017**
Resting O_2 saturation (%)	97 (98–99)	97 (97–98.5)	0.324
Peak O_2 saturation (%)	98 (96–99)	97 (97–98)	0.387
**Pulmonary lung function**			
**DLCO % of predicted**	95 (87–99)	87.3 (81–91)	0.278
**KCO % of predicted**	104 (94–115)	89 (87–92)	0.06
**FEV1 % of predicted**	106 (103–117)	1109.8 (109–114)	0.514
**FVC % of predicted**	112.5 (106–118)	113.8 (111–118)	0.664
**FEV1/FVC (%)**	94 (86–105)	101.5 (90–106)	0.55
**6-MWT distance (meters)**	511 (470–542)	633.5 (615–655)	**0.009**

Values are median (IQR), mean ± SD, or n (%). **Bold** indicates significative differences ($p<0.05$). * Persistent dyspnea was defined as NYHA ≥ II.

Suppl. Table S2. Baseline characteristics and main features during mid-term Figure 19. patients according to the presence of dyspnea in hospitalized patients.

	Persistent Dyspnea* n = 31 (60)	No residual Dyspnea n= 22 (40)	p-value
**Demographics, comorbidities, and symptoms**			
Female sex (%)	22 (71)	9 (29)	**0.029**
Age (years)	59 (54–65)	61 (47–69)	0.342
BSA (m²)	1.77 (1.68–1.94)	1.93 (1.77–2.03)	0.095
Dyslipidemia (%)	6 (19.4)	5 (22.7)	0.999
Hypertension (%)	11 (35.5)	6 (27.3)	0.528
KCCQ summary score	63.4±19.7	80.6±11.9	**0.001**
Fatigue (%)	12 (38.7)	3 (13.6)	**0.046**
Myalgia (%)	1 (3.2)	2 (9.1)	0.563
Neurological symptoms (%)	2 (6.5)	7 (31.8)	**0.025**
**Resting echocardiographic findings**			
LAVI (mL/m²)	22.1 (17.2–31.3)	23.7 (19.4–26.1)	0.748
LVEF (%)	65 (60.5–67.5)	61.5 (59–71)	0.745
Mitral E/A ratio	0.85 (0.76–1.08)	0.83 (0.67–1.06)	0.843
Average E/e′ ratio	7 (5.4–8.9)	6.2 (4.9–7.3)	0.195
TAPSE (mm)	23 (20–27)	23 (22–25)	0.813
Global longitudinal strain (%)	19.5 (21.7–18)	19.6 (21.6–19)	0.992
**Cardiopulmonary exercise test**			
Peak Vo_2 (ml/min/kg)	17.5 (14–19.7)	21.6 (18.4–25)	**0.001**
% of predicted pVo_2	77 (61–89.5)	95 (85–100)	**0.002**
Vo_2 at AT_1 (ml/min/kg)	12.8 (8.7–15.4)	17 (14.5–19.1)	**0.004**
Vo_2 /HR	6.9 (8.4–11)	11.2 (8.4–13.4)	**0.015**
VE/Vco_2 slope	33.1 (30.9–38.3)	28.8 (26.9–31.6)	**0.004**
$PETCO_2$ at AT_1 (mmHg)	34 (32–38)	38 (35–40)	**0.010**
% of predicted HR	87 (77.8–96.1)	95 (87–103)	**0.02**
Resting O_2 saturation (%)	97 (95–97)	97 (96–98)	0.216
Peak O_2 saturation (%)	97 (96–97)	97 (95–98)	0.870
**Pulmonary lung function**			
DL_{CO} % of predicted	84 (70–92.1)	91 (85–102)	**0.018**
K_{CO} % of predicted	93 (83–104.3)	104.3 (91–112)	0.052
FEV1 % of predicted	113 (102–122)	115 (105–124)	0.690
FVC % of predicted	119 (102–134)	118 (104–121)	0.816
FEV1/FVC (%)	99 (87–107)	102 (98–104)	0.657
6-MWT distance (meters)	550 (465–600)	582.5 (537.5–640)	**0.039**

Values are median (IQR), mean ± SD, or n (%). **Bold** indicates significative differences (p<0.05). * Persistent dyspnea was defined as NYHA ≥ II.

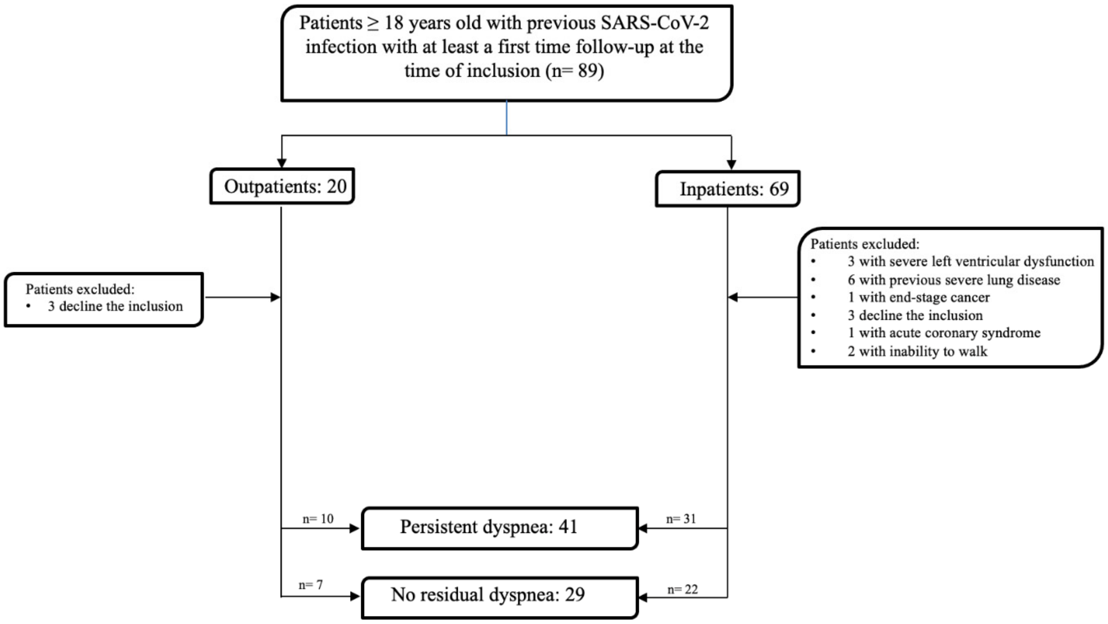

Suppl. Figure S1. Schematic flowchart of the recovered COVID-19 patients included in the study, showing main findings.
